# Anti‐Erythrocyte Antibody Formation in Individuals With and Without Known Sensitisation Pathways Reflecting Indications for Extended Immunohaematology Screening

**DOI:** 10.1002/jcla.70357

**Published:** 2026-09-10

**Authors:** Milanka Milosavić, Marko Lilić, Gordana Guzijan, Dušan Vučetić

**Affiliations:** ^1^ Laboratory for Molecular Immunohematology Institute for Transfusion Medicine of Republika Srpska Banja Luka Bosnia and Herzegovina; ^2^ School of Medicine University of Mostar Mostar Bosnia and Herzegovina; ^3^ Medical Faculty University of Banja Luka Banja Luka Bosnia and Herzegovina; ^4^ Institute for Transfusiology and Haemobiology, Military Medical Academy Belgrade Serbia; ^5^ Faculty of Medicine, Military Medical Academy University of Defense Belgrade Serbia

**Keywords:** alloimmunisation, antibody, blood transfusion, red blood cells

## Abstract

**Background:**

Blood transfusion therapies are of vital importance for many patient groups. They can lead to various complications, with the production of anti‐erythrocyte antibodies (RBC Ab) being of particular importance. To ascertain which group of participants requires greater protection against RBC Abs, we conducted a study on the antibody incidence in part of Bosnia and Herzegovina—Republika Srpska.

**Methods:**

We investigated the yearly incidence of the identified RBC Abs from 2016 until the end of 2022. The identification of RBC Abs was performed using the gel method on various platforms with commercial immunohaematological panels. For statistical analysis, Python 3.12 was used. The groups were formed based on the likely form of the RBC Ab development.

**Results:**

In 1106 participants, a total of 1297 antibodies were detected. All groups of patients showed significance that corresponded to the statistical hypothesis set beforehand, which indicated the influence of additional undefined parameters. In the third group, containing participants with no known history of an immunisation pathway, a significant result was observed for anti‐K (*p* = 1.344 × 10^‐6), which was completely unexpected.

**Conclusion:**

All study groups indicated that it would be advantageous to implement the additional analyses to their routine testing. Further research is needed to identify and prepare prevention strategies for all patient groups from this study.

## Graphical abstract

– The yearly incidence of anti‐red blood cell antibodies stratified by participant groups and temporal evolution.
– Overall significance *p* = 3.756 × 10^−29^.

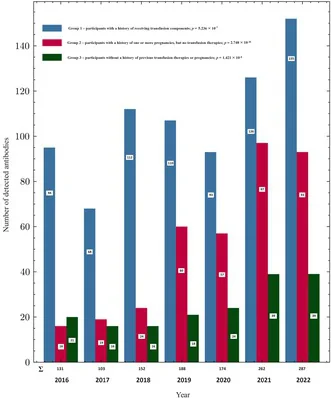

## 1 Introduction

For many years, blood transfusion has remained the only available treatment for numerous patient groups. Since the discovery of blood groups, the development of transfusion medicine has required increasingly complex logistics to ensure the safest possible blood supply.

One of the most important challenges in today's work in transfusion services is achieving better antigenic matching between donors and recipients. The preparation of better‐matched components is becoming increasingly important due to the development of anti‐erythrocyte antibodies (RBC Ab), which have been shown to significantly impact the subsequent treatment of these patients [1].

Along with RBC antigens, the antibodies formed against them have been discovered. However, there still remains a significant gap in understanding the mechanism underlying RBC Ab formation. Although 99% of the population theoretically have the potential to develop RBC Ab during each instance of receiving a blood component or during pregnancy, this is often not the case in practice. The detected prevalence of RBC Ab ranges from 2%–5% in most populations, though it is believed to be even higher [2]. The detection of RBC Ab is a multifaceted process influenced by various factors, including the timing of testing following transfusion, the types of tests used, patient diagnoses, and other clinical and procedural variables [3, 4].

The largest cohort of patients who have developed RBC Abs (responders) has been reported in patients with sickle cell disease, with a prevalence of approximately 40%–50%. ulk of research has been focused on this population of patients [5, 6]. While this particular condition may not be prevalent in the investigated population, many other haematological and oncological diagnoses require blood transfusion therapies irrespective of the presence of developed RBC Abs, thereby making the operational procedures for transfusion services even more intricate.

In the daily routine of the Institute for Transfusion Medicine of Republika Srpska (ITMRS), there has been a noticeable increase in the number of patients with developed RBC Ab over the last couple of years. Addressing the logistical and additional resources required to manage these patients prompted us to investigate the incidence of RBC Abs. The aim of this study was to identify the specificities of RBC Abs and determine which population would require additional immunohaematological testing supported by targeted standard operating procedures in collaboration with other healthcare facilities.

## 2 Materials and Methods

### 2.1 Participants

Data for each year from 2016 to 2022 were evaluated. This retrospective study included all tested samples (patients and voluntary, unrelated blood donors) at ITMRS in Banja Luka, Bosnia and Herzegovina, in which RBC Abs were identified during the respective year. When a participant had any RBC Ab detected prior to the study period (e.g., in 2014), these RBC Abs were excluded from this study. Additionally, if a participant developed RBC Ab of one specificity in 2016 and of another in 2017, we conducted the analysis for both years and counted them as multiresponders (participants with two or more RBC Abs) in 2017. The results of identification where the specificity of RBC Abs could not be determined were labelled as unspecific. If a participant with an unspecific antibody also had a positive autocontrol reaction, they were classified under the category “autoantibody” (autoAb). All pregnant women who developed anti‐D after receiving hyperimmune globulin were excluded from this study. Therefore, all anti‐D cases detected in this study were considered immune‐mediated.

For each examined year, the standard parameters were gathered (such as gender, average age, ABO system blood group, and RBC Ab specificity). The newly identified RBC Abs for each year were observed in three groups based on the likely mode of their occurrence:

- Group 1: participants who received transfusion components prior to RBC Ab identification;
- Group 2: participants who, prior to identification, had been pregnant at some point in their lives, without a history of transfusion therapies;
- Group 3: participants without a history of transfusion therapy or pregnancies (i.e., not exposed to anticipated immunisation pathways).

Statistical analysis was performed for alloantibodies with an average yearly incidence exceeding 10%, multiresponders, and positive autocontrols (autoAb category). We used the fact that anti‐M is a naturally occurring antibody for which we did not expect any fluctuations in incidence across the years. Therefore, anti‐M was included as well, serving as a control for the statistical process.

This study was conducted in accordance with the principles of the Declaration of Helsinki. The protocol was reviewed and approved by the Ethical Committee of the University Clinical Centre of the Republic of Srpska in Banja Luka, Bosnia and Herzegovina (approval number 01–19–133‐2/21).

## 3 Methods

The indirect antiglobulin test (IAT), used to detect the presence of RBC Abs, was performed using the gel method. At ITMRS, the IAT is mandatory for all cross‐match analyses during the preparation of RBC components. It is also required for every new blood donation. Screening and identification testing were performed in accordance with the EDQM guidelines of the European Directorate for the Quality of Medicines & Healthcare (EDQM) [7]. All antibody testing standard operating procedures used were following the processes described in the respective assay's instructions for use as well as the Technical Manual issued by the Association for the Advancement of Blood and Biotherapy (AABB) [8].

For voluntary blood donors, the RBC Ab screening was performed using the QWALYS 3 EVO automated system using HEMASCREEN POOL IgG + IgM (DIAGAST, France) or on the Techno TwinStation instrument using ID‐DiaCell Pool cards (Bio‐Rad Laboratories, Switzerland), or both. For patients, the gel method was used either with Erytra and the 0.8% two‐cell panel kit Serascan Diana 2 (Grifols, Spain) or with the IHA 500 instrument and the two‐cell screening panel ID‐DiaCell I‐II (Bio‐Rad Laboratories), or both.

At ITMRS, the antibody identification was performed manually or via automated platforms, using 11‐cell panels Identisera Diana (Grifols) or ID‐DiaPanel (Bio‐Rad Laboratories). In participants with multiple alloantibodies (multiresponders), various techniques were performed to assist with RBC Ab specificity resolution [8].

Autocontrol and direct antiglobulin tests were also evaluated in the gel methods. With commercial panels, it was not possible to define which antibody or several are responsible for autoantibody positive reactions. In the analysis, it was considered that the positive autocontrol test can also display the presence of autoantibodies not exclusively directed against RBCs.

All anti‐A1 antibodies identified in this study were detected through a stepwise approach. Following an initial discrepancy between forward and reverse ABO typing in direct agglutination—characterised by positive reactions with A1 test RBCs—the participants' RBCs showed negative reactions with anti‐A1 lectin (Anti‐A1 Lectin, Bio‐Rad Laboratories) and positive reactions with anti‐H (Anti‐H, Bio‐Rad Laboratories). Subsequent RBC antibody identification panels were negative.

### 3.1 Statistical Analysis

The programming language used for statistical analysis was Python 3.12 (Python Software Foundation, USA. Python Language Reference, https://www.python.org/). For graphical data presentation, Matplotlib was used (Matplotlib, version v3.8.3, https://matplotlib.org/). Additionally, we used pandas (pandas, version 2.2.1, https://pandas.pydata.org/) and NumPy (NumPy, version 1.26.2, https://numpy.org/) packages for data manipulation and filtering purposes. NumPy was also used for the weighted mean value estimation using the following formula:

$$x_{w}=\left(\sum_{i}w_{i}x_{i}\right)/\left(\sum_{i}w_{i}\right)$$

$x_{w}$ – weighted average.

$w_{i}$ – number of participants/antibody detections in a year.

$x_{i}$ – number of participants/antibody detections for each category.

The *p*‐values were calculated using a chi‐square test conducted across the observed years. In the chi‐square formula, the expected value was the mean value, and the observed value corresponded to the value from the respective year.

The following hypotheses were set:

– Null hypothesis (H0): Antibody detection follows a consistent pattern determined by the yearly weighted average of detection (2016–2022), a constant. This means that the yearly increase in patients and/or the increase in detected antibodies is statistically not significant.
– Alternative hypothesis (HA): Antibody detection is not a constant function and depends on an additional parameter

The statistical significance level was set at 0.01. All *p*‐values < 0.01 confirm the HA, while those ≥ 0.01 confirm the H0. Extremely low *p*‐values were expressed in scientific notation. Such results were definitely lower than 0.01, thereby confirming the HA.

## 4 Results

Between 01.01.2016 and 31.12.2022, 1297 RBC Abs (938 alloantibodies, excluding anti‐M) were detected in 1106 participants with 160 participants being multiresponders (who had at least two to a maximum of four antibodies). No specific RBC Abs were determined in 248 participants. For 212 unspecific antibodies, auto‐control was positive, and they were included in the positive auto‐control category. The other 36 participants were counted in the unspecific antibody category. Table 1 presents the general characteristics of the investigated cohort. During the years examined, the number of blood donations increased from 11,112 in 2016 to 13,220 in 2022, an increase of 18.97% (Table 2).

**TABLE 1:** General characteristics of 1106 study participants who formed antibodies.

Study group	Overall years 2016 to 2022					
	Patients, *N* (%)	Blood donors, *N* (%)	Blood group, *N* (group %)			
			A	B	O	AB
**Group 1:** Participants who received blood components before the development of anti‐red blood cell antibodies, *N* = 623	623 (56.33%)	0	257 (41.25%)	105 (16.85%)	218 (34.99%)	43 (6.90%)
**Group 2:** Participants with one or more pregnancies, without a history of receiving blood components, *N* = 316	316 (28.57%)	0	120 (37.97%)	64 (20.25%)	112 (35.44%)	20 (6.33%)
**Group 3:** Participants without a history of previous transfusion therapies or pregnancies, *N* = 167	92 (8.32%)	75 (6.78%)	74 (44.31%)	18 (10.78%)	63 (37.72%)	12 (7.19%)

*Note:* Each patient group is represented by its unique color. The color scheme has been maintained consistently.

Abbreviation: *N*, number of participants.

**TABLE 2:** Overview of the blood donations and blood donors who formed antibodies, stratified by years 2016–2022.

Year	2016	2017	2018	2019	2020	2021	2022
Annual blood donations, *N*	11,112	12,134	11,691	12,887	10,672	13,117	13,220
Blood donors who formed antibodies, *N*	14	7	12	14	10	10	8
Blood donors who formed antibodies, as share of blood donations, %	0.13%	0.06%	0.10%	0.11%	0.09%	0.08%	0.06%

There were 22 different RBC Ab specificities identified in addition to the unspecific antibodies and autoantibodies. Table 3 presents an overview of these categories stratified by year of detection. The contribution of a specific antibody to the total number of detected antibodies within each year (yearly incidence) was higher than 10% on average among anti‐D, anti‐E and anti‐K, as well as autoantibodies. Upon consulting the information system, we additionally established that 327 antibodies were newly detected throughout the year 2023 (the year after the present study period ended), an increase of 13.94% from 2022.

**TABLE 3:** Yearly incidence of newly detected specific anti‐red blood cell antibodies across the years.

Detected antibodies, yearly, *N* (%)	Antibody specificity	Year							Total per antibody specificity	Yearly incidence average (2016–2022)	*p*
		2016	2017	2018	2019	2020	2021	2022			
	Anti‐A1	2 (1.53)	3 (2.91)	1 (0.66)	4 (2.13)	/	1 (0.38)	1 (0.35)	12	1.71 (1.33)	0.506
	Anti‐D	15 (11.45)	12 (11.65)	24 (15.79)	33 (17.55)	25 (14.37)	42 (16.03)	40 (13.94)	191	27.28 (14.51)	0.911
	Anti‐c	6 (4.58)	2 (1.94)	8 (5.26)	7 (3.72)	10 (5.74)	13 (4.96)	14 (4.88)	60	8.57 (4.36)	0.907
	Anti‐C	8 (6.11)	3 (2.91)	8 (5.26)	9 (4.79)	7 (4.02)	12 (4.58)	11 (3.83)	58	8 (4.50)	0.968
	Anti‐Cw	3 (2.29)	2 (1.94)	3 (1.97)	7 (3.72)	3 (1.72)	7 (2.67)	6 (2.09)	31	4.43 (2.34)	0.955
	Anti‐E	23 (17.56)	21 (20.39)	24 (15.79)	30 (15.96)	32 (18.39)	36 (13.74)	50 (17.42)	216	30.86 (17.03)	0.944
	Anti‐e	3 (2.29)	/	/	3 (1.60)	2 (1.14)	1 (0.38)	/	9	1.28 (0.77)	0.702
	Anti‐K	14 (10.69)	14 (13.59)	19 (12.50)	22 (11.70)	22 (12.64)	39 (14.89)	37 (12.89)	167	23.86 (12.72)	0.989
	Anti‐Kpa	3 (2.29)	1 (0.97)	1 (0.66)	1 (0.53)	/	3 (1.15)	3 (1.05)	12	1.71 (0.81)	0.790
	Anti‐Kpb	1 (0.76)	1 (0.97)	/	/	/	/	/	2	0.14 (0.14)	NA
	Anti‐M	12 (9.16)	12 (11.65)	11 (7.24)	17 (9.04)	18 (10.34)	21 (8.02)	21 (7.32)	112	16 (8.97)	0.919
	Anti‐*N*	/	/	/	1 (0.53)	/	1 (0.38)	1 (0.35)	3	0.42 (0.18)	0.978
	Anti‐S	2 (1.53)	2 (1.94)	1 (0.66)	1 (0.53)	/	1 (0.38)	7 (2.43)	14	2 (1.07)	0.782
	Anti‐Fya	1 (0.76)	2 (1.94)	3 (1.97)	7 (3.72)	6 (3.44)	15 (5.73)	9 (3.14)	43	6.14 (2.96)	0.525
	Anti‐Fyb	3 (2.29)	1 (0.97)	/	/	/	/	2 (0.70)	6	0.86 (0.56)	0.358
	Anti‐Lea	3 (2.29)	3 (2.91)	2 (1.32)	5 (2.66)	3 (1.14)	5 (1.91)	3 (1.05)	24	3.43 (1.90)	0.986
	Anti‐Leb	1 (0.76)	2 (1.94)	1 (0.66)	2 (1.06)	/	2 (0.76)	3 (1.05)	11	1.57 (0.89)	0.948
	Anti‐Jka	1 (0.76)	5 (4.85)	3 (1.97)	4 (2.13)	9 (5.17)	10 (3.82)	12 (4.18)	44	6.28 (3.27)	0.669
	Anti‐Jkb	1 (0.76)	/	/	1 (0.53)	/	/	1 (0.35)	3	0.43 (0.23)	0.926
	Anti‐Lua	3 (2.29)	/	4 (2.63)	3 (1.60)	2 (1.14)	2 (0.76)	3 (1.05)	17	2.42 (1.35)	0.885
	Anti‐P1	/	1 (0.97)	2 (1.32)	1 (0.53)	4 (2.30)	1 (0.38)	4 (1.39)	13	1.42 (0.99)	0.829
	Anti‐G	/	/	/	1 (0.53)	/	/	/	1	0.14 (0.06)	NA
	AutoantibodyUnspecific	25 (19.08)1 (0.76)	14 (13.59)2 (1.94)	25 (16.45)12 (7.89)	27 (14.36)2 (1.06)	28 (16.09)3 (1.72)	38 (14.50)12 (4.58)	55 (19.16)4 (1.39)	21236	30.28 (16.18)5.14 (2.76)	0.9250.093
**Total**		131 (10.10)	103 (7.94)	152 (11.72)	188 (14.49)	174 (13.42)	262 (20.20)	287 (22.13)	1297	185.29	3.756 × 10^‐29

*Note:* The *p*‐values were calculated using a chi‐square test. Significance was set at *p* < 0.01. Percentages in bold show the average yearly incidence higher than 10%.

Abbreviations: *N*, number of antibody detections; NA, not applicable, /, antibody not detected.

The study participants who developed antibodies, stratified by groups and categories across the years, are shown in Table 4.

**TABLE 4:** Participants who developed anti‐red blood cell antibodies stratified by groups and categories.

Overall	Study group	Category	Year							Total	*p*
			2016	2017	2018	2019	2020	2021	2022		
**Participants who developed anti‐red blood cell antibodies** ***N* = 1106**	**Group 1:** Participants who received blood components before the development of anti‐red blood cell antibodies, *N* = 623	Number of participants *n*, (%)	80 (12.84)	54 (8.67)	99 (15.89)	86 (13.80)	80 (12.84)	103 (16.53)	121 (19.42)	623 56.33%	3.134*10^‐5
		Multiresponders *n*, (%)	11 (13.75)	7 (12.96)	13 (13.13)	17 (19.77)	13 (16.25)	19 (18.45)	27 (22.31)	107 17.17%	0.011
		One antibody *n*, (%)	69 (86.25)	47 (87.04)	85 (86.87)	69 (80.23)	67 (83.75)	84 (81.55)	94 (77.69)	516 82.83%	**0.003**
	**Group 2:** Participants with one or more pregnancies, without a history of receiving blood components. *N* = 316	Number of participants *n*, (%)	14 (4.43)	17 (5.38)	19 (6.01)	52 (16.46)	51 (16.14)	86 (27.22)	77 (24.37)	316 (28.57%)	0.001*10^‐37
		Multiresponders *n*, (%)	1 (7.14)	2 (11.76)	5 (26.32)	7 (13.46)	6 (11.76)	8 (9.30)	13 (16.88)	42 13.29%	0.013
		One antibody *n*, (%)	13 (92.86)	15 (88.24)	14 (73.68)	45 (86.54)	45 (88.24)	78 (90.70)	64 (83.12)	274 86.71%	**2.730 × 10^‐20**
	**Group 3:** Participants without a history of previous transfusion therapies or pregnancies *N* = 167	Number of participants *n*, (%)	19 (11.38)	14 (8.38)	14 (8.38)	21 (12.57)	24 (14.37)	36 (21.56)	39 (23.35)	167 15.10%	1.483*10^‐22
		Multiresponders *n*, (%)	1 (5.26)	2 (14.29)	2 (14.29)	0 /	0 /	3 (8.33)	3 (7.69)	11 6.59%	0.403
		One antibody *n*, (%)	18 (94.74)	12 (85.71)	12 (85.71)	21 (100.00)	24 (100.00)	33 (91.67)	36 (92.31)	156 93.41%	**0.497 × 10^‐3**

*Note:* Group 1 was not investigated for previous pregnancies; Groups 1 and 2 consist solely of patients without any blood donors; Group 3 included blood donors but was not limited to them. *N* = number of antibody detections; *n* = number of antibody detection in examined year; P – Pearson's chi‐squared, significance was set at *p* < 0.01; *p* values in bold show the statistically significant result that corresponded to HA. Each patient group is represented by its unique color. The color scheme has been maintained consistently.

Table 5 displays the detected antibodies stratified by groups and categories across the years.

**TABLE 5:** Detected antibodies stratified by groups and categories.

overall	Study group	Category		Examined year							Total (% of 1297)	*p*
				2016	2017	2018	2019	2020	2021	2022		
**Detected anti‐red blood cells antibodies** ***N* = 1297**	**Group 1:** Participants who received blood components before the development of anti‐red blood cell antibodies, N1 = 758	Σ Group 1 *n*, (% of 758)		94 (12.40)	68 (8.97)	112 (14.78)	110 (14.51)	93 (12.27)	126 (16.62)	155 (20.45)	758 (58.44)	5.236*10^‐7
		Unspecific *n*, (% of 21)		1 (1.06)	2 (2.94)	9 (8.04)	1 (0.91)	1 (1.08)	6 (4.76)	1 (0.65)	21 (1.62%)	NC
		Autoantibody *n*, (% of 89)		18 (19.15)	6 (8.82)	16 (14.29)	14 (12.73)	10 (10.75)	7 (5.56)	18 (11.61)	89 (6.86)	0.035
		Anti‐M		4 (4.26)	2 (2.94)	5 (4.46)	3 (2.73)	3 (3.23)	7 (5.56)	4 (2.58)	28 (2.16)	0.117
		Alloantibodies *n*, (% of total)	*Anti‐D*	*11* *(11.70)*	*5* *(7.35)*	*15* *(13.39)*	*11* *(10.00)*	*7* *(7.53)*	*8* *(6.35)*	*13* *(8.39)*	*70* *(5.40)*	0.285
			*Anti‐E*	*19* *(20.21)*	*18* *(26.47)*	*21* *(18.75)*	*25* *(22.73)*	*22* *(23.66)*	*29* *(23.02)*	*35* *(22.58)*	*169* *(13.03)*	0.165
			*Anti‐K*	*11* *(11.70)*	*13* *(19.12)*	*19* *(16.96)*	*18* *(16.36)*	*17* *(18.28)*	*26* *(20.63)*	*25* *(16.13)*	*129* *(9.95)*	0.117
			*other*	*30* *(31.91)*	*22* *(32.35)*	*27* *(24.11)*	*38* *(34.55)*	*33* *(35.48)*	*43* *(34.13)*	*59* *(38.06)*	*252* *(19.43)*	NC
			Σ alloantibodies	71 (11.45)	58 (9.35)	82 (13.23)	92 (14.84)	79 (12.74)	106 (17.10)	132 (21.29)	620 (47.80)	**2.546 × 10^‐7**
	**Group 2:** Participants with one or more pregnancies, without a history of receiving blood components. N2 = 366	Σ Group 2 *n*, (%)		16 (4.37)	19 (5.19)	24 (6.56)	60 (16.39)	57 (15.57)	97 (26.50)	93 (25.41)	366 28.22%	2.748*10^‐36
		Unspecific *n*, (%)		0 (/)	0 (/)	3 (25.00)	1 (8.33)	2 (16.37)	4 (33.33)	2 (16.37)	12 (0.93)	NC
		Autoantibody *n*, (%)		1 (1.82)	5 (9.09)	2 (3.64)	5 (9.09)	6 (10.91)	20 (36.36)	16 (29.09)	55 (4.24)	4.407*10^‐7
		Anti‐M		1 (3.70)	3 (11.11)	1 (3.70)	3 (11.11)	7 (25.93)	7 (25.93)	5 (18.52)	27 (2.08)	0.121
		Alloantibodies *n*, (%)	*Anti‐D*	*4* *(3.42)*	*7* *(5.98)*	*9* *(7.69)*	*22* *(18.80)*	*18* *(15.38)*	*31* *(26.50)*	*26* *(22.22)*	*117* *(9.02)*	1.114*10^‐6
			*Anti‐E*	*2* *(4.65)*	*3* *(6.98)*	*3* *(6.98)*	*5* *(11.63)*	*9* *(20.93)*	*7* *(16.28)*	*14* *(32.56)*	*43* *(3.32)*	**0.006**
			*Anti‐K*	*3* *(10.71)*	*1* *(3.57)*	*0* *(/)*	*4* *(14.29)*	*5* *(17.86)*	*5* *(17.86)*	*10* *(35.71)*	*28* *(2.16)*	0.013
			*other*	*5* *(5.95)*	*0* *(/)*	*6* *(7.14)*	*20* *(23.81)*	*10* *(11.90)*	*23* *(27.38)*	*20* *(23.81)*	*84* *(6.48)*	NC
			Σ alloantibodies	14 (5.15)	11 (4.04)	18 (6.62)	51 (18.75)	42 (15.44)	66 (24.26)	70 (25.74)	272 (20.97)	**2.242 × 10^‐19**
**Detected anti‐red blood cells antibodies** ***N* = 1297**
	**Group 3:** Participants without a history of previous transfusion therapies or pregnancies N3 = 173	Σ Group 3 *n*, (%)		21 (12.14)	16 (9.25)	16 (9.25)	18 (10.40)	24 (13.87)	39 (22.54)	39 (22.54)	173 (13.34)	1.142*10^‐4
		Unspecific *n*, (%)		0 (/)	0 (/)	0 (/)	0 (/)	0 (/)	2 (66.67)	1 (33.33)	3 (0.23)	NC
		Autoantibody *n*, (%)		6 (8.82)	3 (4.41)	7 (10.29)	8 (11.76)	12 (17.65)	11 (16.18)	21 (30.88)	68 (5.24)	**0.001**
		Anti‐M		7 (12.50)	7 (12.50)	5 (8.93)	10 (17.86)	8 (14.29)	7 (12.50)	12 (21.43)	56 (4.32)	0.605
		Alloantibodies *n*, (%)	*Anti‐D*	*0* *(/)*	*0* *(/)*	*0* *(/)*	*0* *(/)*	*0* *(/)*	*2* *(66.67)*	*1* *(33.33)*	*3* *(0.23)*	0.193
			*Anti‐E*	*2* *(40.00)*	*0* *(/)*	*0* *(/)*	*0* *(/)*	*1* *(20.00)*	*1* *(20.00)*	*1* *(20.00)*	*5* *(0.39)*	0.569
			*Anti‐K*	*0* *(/)*	*0* *(/)*	*0* *(/)*	*0* *(/)*	*0* *(/)*	*8* *(80.00)*	*2* *(20.00)*	*10* *(0.77)*	1.344*10^‐6
			*other*	*6* *(21.43)*	*6* *(21.43)*	*4* *(14.29)*	*0* *(/)*	*3* *(10.71)*	*8* *(28.57)*	*1* *(3.57)*	*28* *(2.16)*	NC
Σ alloantibodies			8 (17.39)	6 (13.04)	4 (8.70)	0 (/)	4 (8.70)	19 (41.30)	5 (10.87)	46 (3.55)	0.035

*Note:* Group 1 was not investigated for previous pregnancies; Groups 1 and 2 consist solely of patients without any blood donors; Group 3 included blood donors but was not limited to them. significance was set at *p* < 0.01; *p* values in bold show the statistically significant result that corresponded to HA. Each patient group is represented by its unique color. The color scheme has been maintained consistently.

Abbreviations: *N*, number of antibody detections; *n*, number of antibody detections in the examined year; /, antibody not detected; NC, not calculated; P, Pearson's chi‐squared test.

The average age of the participants was as follows: 63.16 years in Group 1, 50.26 years in Group 2 and 54.54 years in Group 3.

Three of 11 multiresponders from Group 3, in addition to having an anti‐D, also had an anti‐C antibody, while the other eight multiresponders had anti‐M (IgG) with another alloantibody.

## 5 Discussion

In the preparation of this seven‐year retrospective observational study, we were unable to retrieve the relevant data about the exact number of patients who received transfusion therapy. The information system of our Institute could reveal the number of blood components used; thus, it was not possible to calculate the frequency of RBC Abs among participants. Before the result analysis, we assessed the frequency of ABO blood groups in the participants throughout the study years. The distribution was generally consistent with data from neighbouring Croatia [9]. Since ABO antigens do not affect the development of non‐ABO RBC Ab, this served as confirmation that the study participants' data from each year were suitably comparable.

Although the population in the entity Republika Srpska of Bosnia and Herzegovina is steadily declining due to emigration, the demand for transfusion therapies is increasing each year. The number of blood donations at ITMRS in the investigated period showed a slight increase, which does not explain the increase in the number of participants with developed RBC Abs by 109.73%, from 131 in 2016 to 287 in 2022 (Table 3), *p* < 0.01. It is noteworthy that we observed an additional 13.94% increase in the number of developed antibodies between the last year of this study period (287 in 2022) and the following year (327 in 2023). This fact strengthens the HA proposed in this study. However, the share of the blood donors who formed antibodies remained fairly constant. Individual analysis of specific RBC Ab prevalence did not show a significant rise through the years (Table 3), but there was a clear upward trend in the number of RBC Abs detections in all studied groups, supporting that additional parameters have been affecting antibody detection (Tables 4 and Tables 5).

The participants who received blood components prior to the development of RBC Abs exhibited a significant increase in RBC Abs, aligning with the HA. Alongside a slight rise in administered blood components, this could partially be attributed to the observed growing population of haematological and oncological patients, who are at a higher risk of developing antibodies [10]. These results have additionally shaped the need to look for better tools for targeted extended testing. In a Dutch study, the redefined MINimize Relative Alloimmunization Risks (MINRAR) model for component selection, albeit in a single hospital, demonstrated excellent outcomes in reducing RBC Abs prevalence [11]. For patients at elevated risk of developing RBC Abs and for whom preventing antibody development is paramount for future treatments, this model is under consideration for future implementation in our Institute. It is recommended not to overlook and also consider the impact of HLA molecules, as reflected in the previous study from our own population, and to improve prevention for patient groups most vulnerable to developing RBC Abs [12].

In Group 2, consisting of participants with previous pregnancies, we obtained highly intriguing findings. A study from Serbia reported an elevation in the prevalence of anti‐D among pregnant women, which was similarly corroborated by our findings [13]. The increase in anti‐D can partially be related to the lack of prophylaxis during the wartime conflict in our country (1992–1995), as well as the subsequent detection of developed anti‐D in many participants upon reaching menopause. It has long been established that the K antigen from the Kell system forms on precursor cells and triggers haemolytic disease of the foetus and newborn (HDFN) [14]. A large 2024 study by Sugrue et al. showed a significant increase in high‐risk antibodies for HDFN, where 67% of anti‐K patients had a critically high titre [15]. Interestingly, the present study demonstrated a borderline significant increase in the anti‐K yearly incidence, further supported by the fact that in our healthcare facilities, the postpartum IAT is exclusively conducted on D‐negative mothers. This suggests that the anti‐K could be even more frequent. The same assertion applies to the anti‐E (*p* = 0.006), which also poses a risk of HDFN [16]. For these three alloantibodies, as well as multiresponders, the results of this study clearly advocate for the likelihood of additional parameters having been incorporated into the routine practice. It is established that numerous medications can be integrated into the erythrocyte membrane (codeine, chlorpromazine, pyrimethamine and several others), but their impact on the immune system remains uncertain [17, 18, 19, 20]. This, combined with the increasing use of diverse therapies during pregnancies, including artificial insemination and other interventions, underscores the importance of careful consideration and monitoring [21]. The increase in the number of participants with positive autocontrols also suggests the introduction of additional parameters (Table 5), possibly influenced by the growing incidence of autoimmune disorders observed in many populations, particularly following the COVID‐19 pandemic period [22].

The equally intriguing results demonstrated a significant increase in RBC Abs within Group 3, participants with no history of transfusion therapies or pregnancies prior to the RBC Ab development. Analysis of the data provided in Tables 2 and Tables 5 indicates that the patient population, rather than the blood donors, accounts for the increase in the number of identified RBC antibodies in this study. In this group, anti‐M proved to be a useful control for the other results, as well. There was no significant difference observed for the yearly incidence of anti‐D and anti‐E in the present study. Studies have already reported the presence of these RBC Abs in healthy donors [23]. The authors acknowledge the limitations of this study, specifically the potential for incomplete reporting of previous pregnancies within Group 3, as well as the possibility that patients may have received blood transfusions abroad for which data is unavailable. This may account for the number of multiresponders within this group.

The autoAb results of Group 3 are similar to those of Group 2 (women with previous pregnancies), suggesting a likely shared cause of this increase. A growing number of studies point to an increase in autoimmune disorders associated with COVID‐19 [24, 25]. However, in this group there is an entirely new occurrence of anti‐K in the years 2021 and 2022, clearly indicating an additional parameter influencing their development (*p* < 0.01). The study from southeastern Serbia reported anti‐K as the most prevalent alloantibody in blood donors, which was not seen in earlier studies [26]. Dong L. et al. suggested that the Type 1 interferon immune response to Toll‐like receptor agonists or infection in transgenic mice regulated the development of RBC Abs, with anti‐K antibodies being notably affected by influenza exposure [27]. It is also possible to observe this phenomenon through molecular mimicry. It can be debated whether the anti‐K reactivity detected in this study stems as a response to a genuine K antigen or whether it only mimics it. Nevertheless, for the routine preparation of RBC components for patients, additional tests should be performed and the presence of the K antigen on the unit RBCs should be excluded where necessary. The precise origin of participants' exposure to antigen K remains uncertain. There is no uncertainty that the data for previous pregnancies was correct, as this subgroup consisted of a total of nine males and one female. Nevertheless, we can highlight the need to examine this situation in a larger population.

## 6 Conclusion

One of the future strategies to reduce the incidence of RBC Abs at ITMRS should be matching the prepared blood components for the antigens within the Kell and Rh blood group systems. The strategy of implementing mandatory IAT for all postpartum women, regardless of the D antigen presence, as well as monitoring their pregnancies, could provide new guidelines for protecting this population and likely reveal a higher incidence of antibodies than currently observed. The objective for ITMRS must be to implement electronic crossmatch, that includes extensive RBC phenotype testing for all blood donors and patient samples, thereby facilitating the timely identification of an appropriate unit for each individual patient. Transfusion services are undoubtedly facing increasing challenges in their future work, both in preparing blood components for patients and in managing the increasingly complex logistics involved in these processes.

## Author Contributions

M.M. – conceptualization; M.M. and G.G. formal analysis; M.L., M.M., M.L. and D.V. investigation; M.M., M.L., D.V. and G.G. visualization and wrote the paper.

## Funding

The authors have nothing to report.

## Ethics Statement

This study protocol was reviewed and approved by the Institutional Ethical Committee, University Clinical Centre of the Republic of Srpska in Banja Luka, Bosnia and Herzegovina, approval number 01–19–133‐2/21.

## Conflicts of Interest

The authors declare no conflicts of interest.

## Data Availability

The data that support the findings of this study are available on request from the corresponding author. The data are not publicly available due to privacy or ethical restrictions.
